# Safety of long-term electrical peripheral nerve stimulation: review of the state of the art

**DOI:** 10.1186/s12984-018-0474-8

**Published:** 2019-01-18

**Authors:** Clara Günter, Jean Delbeke, Max Ortiz-Catalan

**Affiliations:** 1 Biomechatronics and Neurorehabilitation Laboratory, Department of Electrical Engineering, Chalmers University of Technology, 41296 Gothenburg, Sweden; 2 LCEN3, Department of Neurology, Institute of Neuroscience, Ghent University, C. Heymanslaan, 10, 9000 Ghent, Belgium; 3 Integrum AB, Krokslätts Fabriker 50, 43137 Mölndal, Sweden

**Keywords:** Electrical stimulation, Safety, Peripheral nervous system, Nerve stimulation, Implants

## Abstract

**Background:**

Electrical stimulation of peripheral nerves is used in a variety of applications such as restoring motor function in paralyzed limbs, and more recently, as means to provide intuitive sensory feedback in limb prostheses. However, literature on the safety requirements for stimulation is scarce, particularly for chronic applications. Some aspects of nerve interfacing such as the effect of stimulation parameters on electrochemical processes and charge limitations have been reviewed, but often only for applications in the central nervous system. This review focuses on the safety of electrical stimulation of peripheral nerve in humans.

**Methods:**

We analyzed early animal studies evaluating damage thresholds, as well as more recent investigations in humans. Safety requirements were divided into two main categories: passive and active safety. We made the distinction between short-term (< 30 days) and chronic (> 30 days) applications, as well as between electrode preservation (biostability) and body tissue healthy survival (harmlessness). In addition, transferability of experimental results between different tissues and species was considered.

**Results:**

At present, extraneural electrodes have shown superior long-term stability in comparison to intraneural electrodes. Safety limitations on pulse amplitude (and consequently, charge injection) are dependent on geometrical factors such as electrode placement, size, and proximity to the stimulated fiber. In contrast, other parameters such as stimulation frequency and percentage of effective stimulation time are more generally applicable. Currently, chronic stimulation at frequencies below 30 Hz and percentages of effective stimulation time below 50% is considered safe, but more precise data drawn from large databases are necessary. Unfortunately, stimulation protocols are not systematically documented in the literature, which limits the feasibility of meta-analysis and impedes the generalization of conclusions. We therefore propose a standardized list of parameters necessary to define electrical stimulation and allow future studies to contribute to meta-analyses.

**Conclusion:**

The safety of chronic continuous peripheral nerve stimulation at frequencies higher than 30 Hz has yet to be documented. Precise parameter values leading to stimulation-induced depression of neuronal excitability (SIDNE) and neuronal damage, as well as the transition between the two, are still lacking. At present, neural damage mechanisms through electrical stimulation remain obscure.

## Introduction

Despite numerous applications and the reported benefits of peripheral nerve stimulation, descriptions of specific safety requirements remain scarce. Agnew and McCreery, who authored a number of papers on safety aspects of electrical stimulation of peripheral nerves in cats, reviewed and summarized their findings almost three decades ago [1]. The literature on safe, long-term, and continued stimulation of peripheral nerves has grown marginally since then. More recent reviews have addressed general safety aspects of neural stimulation with particular focus on electrochemical processes [2, 3], mainly with regards to stimulation of the central nervous system [4].

Various applications require direct peripheral nerve stimulation. Prostheses involving motor nerve stimulation include respiratory ventilation [5] and correction of foot drop [6]. More recently, promising results have been obtained stimulating afferent fibers for prosthetic sensory feedback in acute [7–16] and chronic experiments [17–22].

Safety aspects of electrical stimulation are particularly crucial in chronic applications. Safety considerations range from the assessment of biostability (including passive electrode preservation and maintained functionality despite the implanted tissue reaction) and harmlessness through the identification of acceptable limits for various electrical stimulation parameters. Animal experiments raise questions about inter-species transferability. Even within the same species, results cannot always be extrapolated from one tissue to the other (central versus peripheral nervous system). Furthermore, within the peripheral nervous system, different nerve fibers conduct action potentials at different speeds [23], and variable frequency and duration [24], which can affect their tolerance to electrical stimulation regimes. On the basis of an overview of recent literature, we aim at inferring essential safety rules governing peripheral nerve stimulation in humans.

## Passive safety

Various types of highly sophisticated and specialized electrodes have been developed for interfacing with the nervous system. Here, we classify electrodes in terms of size, material, and electrochemical properties, as well as local safety aspects in terms of biostability and harmlessness. Issues not specific to electrodes such as toxicity of leaching chemicals, infection risks, and surgery-linked side effects were not incorporated in this review.

### Structural classification

**Fig. 1:**
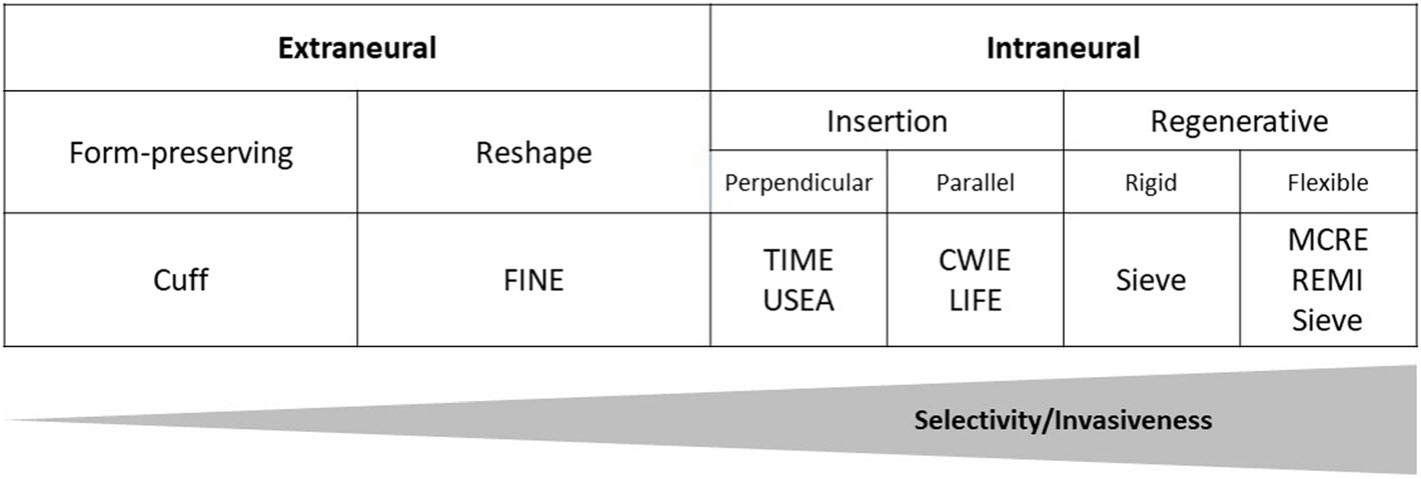
Classification of electrodes used in neurostimulation. Flat Interface Nerve Electrode (FINE), Transversal Intrafascicular Multichannel Electrode (TIME), Utah Slanted Electrode Array (USEA), Coiled Wire Intra-neural Electrode (CWIE), Longitudinal IntraFascicular Electrode (LIFE), Micro-/MultiChannel Roll Electrode (MCRE), Regenerative MultiElectrode Array (REMI)

Interaction of electrodes can be expected to be different according to the tissue component in contact (epineurium, perineurium, and endoneurium). The formation of a fibrous encapsulation can modify this situation and result in functional changes over time. As suggested in a review by Kim and Romero-Ortega [25], electrodes used in peripheral nerve stimulation can be broadly classified as extra- or intra-neural, before being further divided in subcategories (Fig. 1).

Extraneural electrodes are applied outside the epineurium. They may allow the nerve to remain essentially in its original shape, as with cuff electrodes [26], or have selectivity-improving reshaping characteristics, as with the flat interface nerve electrodes (FINE) [27]. The more invasive intraneural electrodes penetrate the epineurium and can be further sub-divided into insertable or regenerative electrodes. Insertable electrodes may be implanted perpendicular or parallel to the nerve axis [28–30]. A further division into extra- and intra-fascicular electrodes is possible as their resting location would have a non-negligible effect on electrical stimulation, albeit fibrous tissue encapsulation of the electrode over time can make such distinction challenging. Regenerative electrode implantation includes an initial neural fiber transection followed by regeneration through regrowth. Rigid or flexible substrate designs have been proposed for this purpose [31–35].

The choice of electrode involves a trade-off between selectivity and invasiveness. Intraneural electrodes are expected to be more advantageous when considering selectivity due to the short distance to target, whereas extraneural electrodes are less invasive but also less selective.

Data collected by Navarro et al. point to the fact that currently in clinical practice, implanted electrodes are generally extraneural [36]. Presently, with regards to long-term human implantations, extraneural electrodes still emerge as the most favorable choice for clinical applications [26, 37].

### Electrical classification

In electrical terms, an electrode can roughly be described as a capacitor in parallel with a resistor. These model components vary for different contact interfaces. Electrodes can be classified as either ideally polarizable or ideally non-polarizable. An ideally polarizable electrode behaves predominantly as a capacitor and has a large resistive component, whereas the resistive component of ‘ideally non-polarizable electrodes’ is small enough for the capacitive component to be ignored. Correspondingly, the charge transfer at the electrode-electrolyte-interface is characterized as being capacitive (non-faradaic) or faradaic. A faradaic charge transfer involving ionic exchanges with the electrolyte through oxidation or reduction reactions is non-reversible unless the electrode is built in such a way that the reactions can continue indefinitely. A commonly used electrode of this type is the silver/silver-chloride electrode. Unfortunately, it cannot be implanted due to toxicity.

The capacitive charge transfer characterizing rapidly polarizable electrodes refers to a redistribution of local charges without electrochemical exchange. Within a very limited range, these capacitive processes are reversible. Some metals, such as platinum, exhibit a property named pseudocapacitance whereby, although a faradaic reaction occurs and electrons are effectively being transferred, the resulting products remain bound to the electrode surface and thus available to undo the chemical reactions. Hence, these reactions are also reversible.

Irreversible reactions must be avoided as their product accumulation will end up being harmful to the local tissues and/or to the electrode efficiency. This is of course a major issue for chronically implanted electrodes. The importance of using capacitive or pseudocapacitive processes to ensure reversibility of reactions was emphasized by Brummer and Turner in 1977 [38]. A more detailed description of electrochemical processes and electrical circuit models can be found in recent reviews [2, 4]. Methods such as cyclic voltammetry, impedance spectroscopy, and voltage transients [3] allow to characterize the electrochemical behavior of electrodes.

### Biostability and harmlessness

The word ‘biocompatibility’ is often used ambiguously in the context of electrodes, referring to completely different issues. One meaning of the expression, which we shall refer to as ‘harmlessness’, describes how well the living organism tolerates and survives the implant without triggering unacceptable reactions or changes. We suggest limiting the concept of ‘biostability’ to designate the device resistance to the biologic medium and its ability to remain chronically functional after implantation. ‘Biocompatibility’ sums these aspects together and therefore is a requirement for any implantable device.

Harmlessness and biostability should be considered separately. Both these characteristics must be evaluated in active implanted devices as well as in the passive mode when the implant is idle (see Table 1). For example, corrosion is certainly activated by stimulation but can also be present at rest. Not only can this impair electrode function, but it also raises concerns about harmlessness; electrochemical by-products of corrosion can be toxic or trigger an exaggerated local inflammatory reaction. These distinctions are necessary to ensure that all potentially harmful aspects are addressed. Combining observations to conclude in terms of ‘acceptable’ or ‘not acceptable’ is not contributive to progress about these matters. Finally, one should not only consider the implanted electrode and the electrical stimulation, but also what can be called harmlessness of the therapy, looking at the effects of the therapy itself. The induction of damaging physiologic overload is an example of such a situation.

**Table 1:** Biocompatibility, biostability, and harmlessness

Biocompatibility	
Biostability	Harmlessness
The electrodes and their functionality	The damage to the organism
Passive	Idle conditions (no stimulation)
Active	Working conditions (stimulation)

In chronic neural stimulation of various peripheral nerves in humans, extraneural electrodes have proven biostable in the peroneal nerve for at least 12 years [6] in passive and active testing. Similar compatibility was shown for up to 18 years stimulating the phrenic nerve [5]; up to 8 years stimulating the median, radial, sciatic, and ulnar nerves [39]; and up to 11 years stimulating motor and sensory nerves in a feedback controlled application [26]. Similarly, our research group has employed self-sizing spiral cuff electrodes to provide sensory feedback in prosthetic hands used outside the laboratory in daily-life activities [22] with no signs of neural damage or electrode deterioration after 18 months of uninterrupted use.

Most often, electrodes implanted on peripheral nerves are made of Platinum-Iridium and Silicone. The passive biostability of the materials used to construct the electrode and leads are often determined from the documented use of the same products in other applications. Numerous studies have confirmed passive biostability of various electrode types. Similarly, local tissue reaction or tissue health after implantation has been mostly well-investigated. In terms of biological response to the electrode, Merill et al. has developed a classification of tissue reactions to different materials [2]. Various biological responses to implanted materials were also discussed in depth by Anderson [40].

However, during surgery, peripheral nerves are sensitive to stretching [41], blood toxicity, and drying out [42]. They must therefore be handled most delicately during implantation. Initial issues with implantable extraneural electrodes often result from mechanical constriction and injury to the nerve during the surgical implantation process [43, 44]. Mechanical damage can also occur later. Electrode design evolved toward easy-to-apply constructions such as the Huntington helical electrode (used in e.g. [45]). Additionally, newer and more flexible spiral cuff electrodes [46, 47] leave room for nerve swelling immediately after implantation while still maintaining a snug fit for good electrical contact, and for preventing movement-induced injury [26]. Cylindrical cuff electrodes with elastic flaps have also been suggested as suitable in this regard by Loeb and Peck [48]. In addition to surgical challenges posed by electrode application, specific mechanical stress factors must be considered [49].

Intramuscular and epimysial electrodes seem to induce tissue responses similar to those of extraneural electrodes, including tissue encapsulation [50, 51]. Grill and Mortimer conducted research on tissue response to chronic implantation of extraneural electrodes [52] and on the input-output properties in terms of specific electrode current and the generated torque as a method to characterize selectivity [51]. After chronic implantation of spiral cuff electrodes around the cat sciatic nerve, as a foreign body reaction, they found connective tissue encapsulating the electrode in all cases. The neural tissue appeared healthy proximal to the cuffs, but showed moderate morphological changes at cuff level and somewhat more pronounced distal to the cuff in some cases [52]. With respect to input-output properties, Grill and Mortimer showed that the stimulus current amplitude required to generate a specific torque varies immediately after implantation, but becomes chronically stable after a period of 8 weeks. They assumed that this evolution mainly reflects the formation of fibrous tissue [51]. It was, however, not possible to find a direct correlation between the functional and morphological changes observed. This was attributed to a low sensitivity of the measurement parameters, as well as several additional factors influencing the outcome, such as muscular hypertrophy compensating for loss of some motor units [52].

Girsch et al. and Larsen et al. showed that neural damage induced by the implantation and presence of an extraneural electrode is reversible [53, 54]. Girsch et al. assessed nerve lesions in the rat sciatic nerve 10 days, 3 weeks, and 3 months after cuff electrode implantation, and noted a gradual recovery from 4.74 to 0.57% of altered fascicular area. Larsen et al. evaluated axonal loss in the rabbit tibial nerve 2 weeks and 16 months after implantation of cuff electrodes, and demonstrated full regeneration after an initial loss of 27% of myelinated axons.

Concerning intraneural electrodes, Christensen et al. investigated the response to a Utah Slant Electrode Array (USEA) in the cat sciatic nerve and found strong tissue reactions, such as persistent active inflammation, even 22 to 26 weeks after implantation [55]. Lago et al. assessed the harmlessness of different longitudinal intrafascicular electrodes (LIFE), namely thin-film (tf-LIFE) and platinum (Pt-LIFE), in the rat sciatic nerve. For both electrode types they found functional deficits (significantly longer latency) in the implanted nerve after 30 days, but recovery after 60 and 90 days. However, in some cases, significant reduction in amplitude of the compound muscle action potential and compound nerve action potential lasted for the entire duration of the study (90 days for Pt-LIFE electrodes and 30 and 60 days for tf-LIFE electrodes) [56]. Rossini et al. observed a steady increase in threshold using thin-film based intrafascicular electrodes (tf-LIFE4) in human median and ulnar nerves until stimulation at a safe charge level became impossible [10]. They attributed this to fibrotic tissue reaction and accommodation effects. There is at present no report on chronic use of intraneural electrodes in humans. However, the USEA and LIFEs have been used successfully in short-term (less than a month) experiments in humans [7–9, 12, 14].

## Active safety – Stimulation parameters

**Fig. 2:**
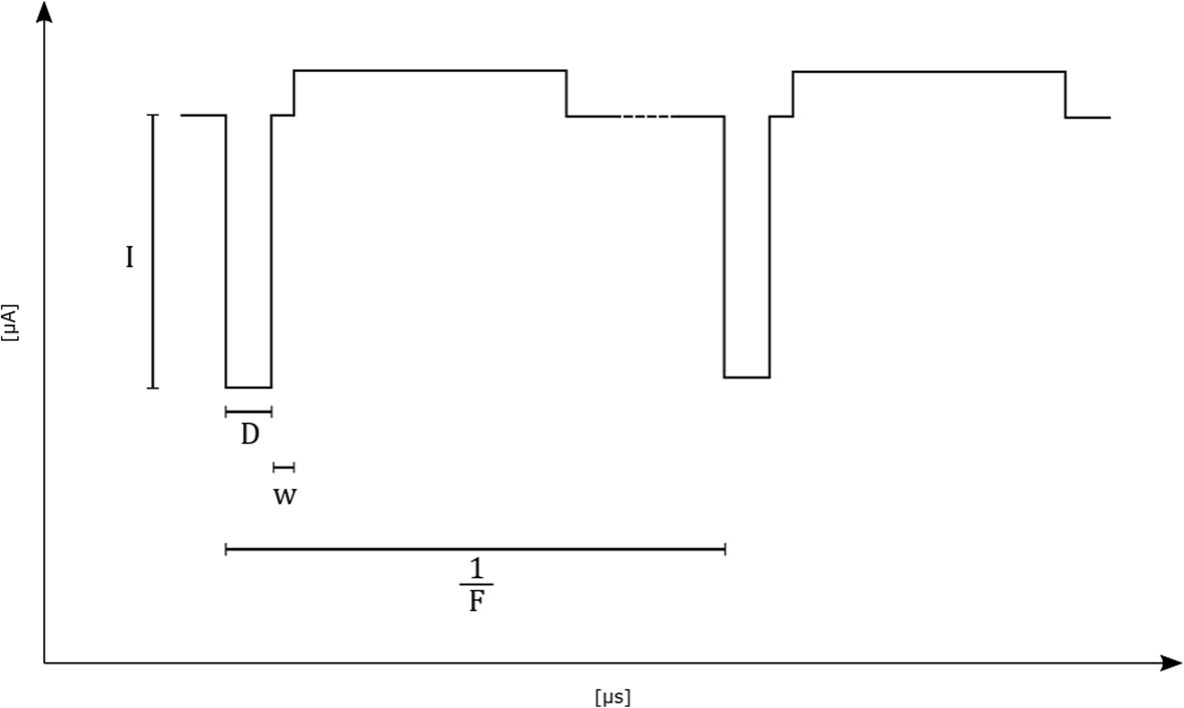
Biphasic, asymmetric, square pulse with inter-phase delay. Current of stimulating phase of stimulation (I), duration of stimulating phase of stimulation (D), inter-phase delay (w), stimulation pulse frequency (F)

Stimulation parameters must be selected carefully, considering electrode corrosion and tissue damage caused by passage of electric charges, as well as physiologic aspects such as stimulation efficiency and tolerance to the therapy. Generally, controlled current pulses are preferred over controlled voltage pulses, although some early attempts were made with controlled voltage stimuli [57]. As long as they do not saturate, current controlled stimulators are immune to variations in electrode impedance. Parameters discussed here include charge injection, pulse amplitude, pulse width, pulse shape (including phase duration, separation, and phase ratio of bi-phasic pulses), pulse frequency, train rate, train duration (=‘ON’ period), stimulus cycle (=‘ON’ + ‘OFF’) period, percentage of effective stimulation time, stimulation application time, and treatment duration. A common bi-phasic stimulus pulse is displayed in Fig. 2, and a summary of parameters, labels, and units used in this work can be found in Tables 2 and 3.

**Table 2:** Standard stimulation parameters

Label	Parameter	Unit
A	Electrode surface area	cm^2^
D	Duration of stimulating phase of a stimulus pulse	µs
D'	Duration of reversal phase of a stimulus pulse ^a^	µs
F	Stimulation pulse frequency	Hz
I	Current of stimulating phase	µA
I'	Current of reversal phase ^a^	µA
N	Number of pulses per train	Unitless
R	Train Rate	Hz
w	Inter-phase delay ^a^	µs
P	Total application time (sum of the ‘ON’ and ‘OFF’ periods) per uninterrupted treatment duration	Hours
S	Effective stimulation time (= sum of the ‘ON’ periods) per uninterrupted treatment duration	Hours
Y	Treatment duration/Implantation Time	days OR months OR years

^a^ if applicable

**Table 3:** Derived stimulation Parameters

Label	Derivation	Parameter	Unit
c	$\mathrm{I}/{\mathrm{I}}^{\hbox{'}}$	Amplitude of stimulating and reversal phase ratio	unitless
O	$\mathrm{S}/\mathrm{P}$	Percentage of effective stimulation time	%
Q	*I* ∙ *D*	Charge of stimulating phase	nC
QD	$\left(\mathrm{I}\bullet \mathrm{D}\right)/\mathrm{A}$	Charge of stimulating phase per unit area	nC/cm^2^
T	$\mathrm{S}/\mathrm{P}$	Train duration	s
wF	*F* ∙ *O*	Weighted frequency of effective stimulation	Hz

Excitable cells such as nerve or muscle cells can be activated by electric current inducing depolarization (i.e., a reduction or even inversion of the resting voltage across the cell membrane). Upon reaching a given threshold, the depolarisation triggers self-reinforcing mechanisms. The result is an action potential that is a short-duration membrane potential inversion able to induce the same phenomenon in neighbouring membrane regions. The action potential event thus propagates along the excitable fiber. Due to the electrical capacitance of cell membranes, the stimulation pulse duration affects the current required to reach the threshold voltage for membrane activation. Therefore, excitability of a tissue is commonly characterized by a strength-duration curve, representing the current amplitude required to reach a threshold with pulses of a given duration. Two parameters characterize this curve: the rheobase current and the chronaxie time. The rheobase is defined as the minimal current required to evoke an action potential with an infinitely long duration pulse, and the chronaxie is the minimum duration to evoke an action potential with pulse intensities of twice the rheobase current [58]. Excitation properties of different tissues are thus quantified by their specific strength-duration characteristic first experimentally derived in the early 1900’s by Weiss [59] and Lapicque [60]. The non-linearity of these curves explains why electric charge injection required to reach threshold tends to increase with pulse duration, especially above the chronaxie value.

### Charge

The level of electrode corrosion, an irreversible reaction, is primarily related to charges faradaically and irreversibly transferred. For different electrode materials, charge injection limits were defined by Robblee and Rose based on the reversibility of the resulting processes [61]. However, relationships between stimulation pulse parameters and tissue damage are more obscure. McCreery et al. reported similar levels of neural damage to a cat’s cortex for faradaic and capacitive electrodes [62], but noted that their stimulus waveform was ideal for the faradaic electrodes used.

Safety suggestions based on charge injection per phase (pulse amplitude times pulse width per phase) and charge density (pulse amplitude times pulse width per phase over electrode surface area) have been reported in various contexts in the past [4, 63–65]. Shannon derived a model for safe stimulation [65] largely based on experimental data by McCreery et al. [64]. He defined the boundary for safe stimulation with the equation as:

$$ \log (QD)=k-\log (Q) $$

where QD is the charge density per phase in nC/cm^2^/phase, Q is charge per phase in nC/phase, and k should be selected between 1.5 and 2.0 [65], with a k-value of 1.5 to 1.8 being most common [4]. Breaking down this equation, a k-value of 1 would result in charges strictly proportional to the electrode area, whereas a k-value of 2 would result in charge strictly proportional to the electrode perimeter. The safe current limit is thus not strictly proportional to the area, as often implied by using expressions of current or charge/unit area. The electrode perimeter is often a better normalization reference but comparing limits for electrodes of different sizes remains questionable. This issue results from the ‘edge effect’: the non-uniform current distribution at the surface of an electrode. This geometrical problem has been discussed in several publications [66–74].

Geometric factors not only involve the electrodes but also the structure of the tissue being stimulated. Stimulating nerve fibers that are activated at the nodes of Ranvier cannot directly be compared with micro-electrodes directly on the axon or cell membrane. Cogan et al. discussed the differences in stimulation thresholds based on electrode size, for instance, differences between macro- and microelectrodes [4]. The popular 30 μC/cm^2^ charge density threshold considered for macroelectrodes becomes a 4 nC/phase (charge per phase) threshold when dealing with microelectrodes, that is when electrode surface area can be neglected for sufficiently small surfaces. Maximal current or charge density cannot be based solely on so-called reference values unless all other conditions are equal, which is rarely the case. It is also important to point out that most recommendations have been established for brain stimulation while the corresponding literature about peripheral nerves remains scarce.

### Duration

The safety of acute and chronic peripheral nerve stimulation has been demonstrated in animals and humans for pulse durations from 1 to 300 μs for the purpose of motor function and sensory feedback [43, 44, 75] (see Table 4). Longer pulse durations up to 500 μs have been reported in chronic vagus nerve stimulation without signs that raised safety concerns [76]. In regards to biostability, Mortimer et al. observed an increased rate of electrode failure with longer pulse durations from 200 μs to 500 μs, and associated this observation with electrode corrosion [77]. Similarly, Merrill et al. noted that a narrow pulse is desirable to decrease occurrence of electrochemical reactions, and therefore reduce electrode degradation [2]. These findings indicate that when technically feasible, pulses of short duration minimize charge displacements and therefore corrosion.

**Table 4:** Examples of stimulation parameters in literature

Publication	Subjects	Electrode type	Stimulation/ Recording	Electrode Placement	Treatment Duration (Y)	Purpose of Stimulation	Stimulation Parameters											
							Charge of Stimulating Phase (Q)	Current of Stimulating Phase (I)	Duration of Stimulating Phase (D)	Stimulation Frequency (F)	Train Duration (T)	Effective Stimulation Time per Day (S)	Potential Stimulation Time per Day (P)	Percentage of Effective Stimulation (O)	Waveform			
															Shape	Polarity	Symmetry	Inter-Phase Delay (w)
Electrical Stimulation for Sensory Feedback																		
Clippinger et al. [57]	A	Cuff	S	M	< 2 y	S	–	^c^	–	0–100	–		–	–	–	–	–	–
Anani et al. [97]	AB	Needle	S	M, R	< 1 d	S	< 200	250–2600	30–250	2.5–160	1	–	–	20	Rect.	M	–	–
Walker et al. [115]	A	Cuff	S	M	3 y	S	–	^c^	20–640	0–90	–	2	24	8.3	–	–	–	–
Anani and Körner [116]	AB	Needle	S	forearm	< 1 d	S	–	^b^	200	10–80	120	–	–	–	Rect.	^a^	–	–
Anani and Körner [116]	A	Needle	S	M, R, U	< 1 d	S	40–300	400–3000	100	10–80	–	–	3–4	–	Rect.	^a^	–	–
Ochoa and Torebjörk [95]	AB	Needle	S, R	M, U	< 1 d	S	–	^c^	250	1–300	2	–	–	3–6	Rect.	M	–	–
Dhillon et al. [8]	A	LIFE	S, R	M, U, P	< 1 m	S	< 50	< 200	250	10–800	0.5	–	–	–	Rect.	M, B	A, S	–
Dhillon and Horch [7]	A	LIFE	S, R	M	< 1 m	S	–	^b^	300	10–500	0.5	–	–	–	–	–	–	–
Dhillon et al. [9]	A	LIFE	S, R	M	< 1 m	S	< 60	1–200	300	10–510	0.5	–	–	–	–	B	–	–
Rossini et al. [10]	A	Tf-LIFE4	S, R	M, U	< 1 m	S	< 3	10–100	10–300	10–500	0.3–0.5	–	–	–	Rect.	–	–	–
Horch et al. [11]	A	LIFE	S	M, U	< 2 w	S	–	^b^	290	20–200	–	–	–	–	–	B	–	75
Clark et al. [12]	A	USEA	S, R	M, U	< 1 m	S	2–2.4	10–12	200	200	0.2	–	–	–	–	–	–	–
Ortiz-Catalan et al. [18]	A	Cuff	S	U	20 m	S	–	100–180	–	8–30	–	–	–	–	–	B	–	–
Raspopovic et al. [13]	A	TIME	S	M, U	< 1 m	S	< 24	240	100	50	0.5	–	–	–	Rect.	B	–	–
Tan et al. [17]	A	FINE	S	M, R, U	16 m & 24 m	S	–	< 2000	20–167	1–1000	< 60	–	4–6	–	Rect	B	A	–
Davis et al. [14]	A	USEA	S, R	M, U	< 1 m	S	0.2–20	1–100	200	1–320	0.2–60	–	2	–	–	B	–	100
Graczyk et al. [19]	A	FINE	S	M, R, U	3 y & 4 y	S	–	^b^	< 255	12.5–166	1–5	–	–	–	Rect.	B	–	–
Oddo et al. [15]	A	TIME	S	M	< 1 m	S	16	160	100	–	–	–	–	–	–	–	–	–
Schiefer et al. [21]	A	FINE	S	M, R, U	2 y & 3 y	S	–	–	255	10–125	–	–	5–6	–	–	B	–	–
Mastinu et al. [20]	A	Cuff	S	U	4 y	S	–	–	–	< 30	–	–	–	–	Rect.	B	A	50
Ortiz-Catalan et al. [22]	A	Cuff	S	U	4 y	S	–	–	–	–	–	–	–	–	–	–	–	–
Wendelken et al. [16]	A	Cuff	S, R	M, U	4–5 w	S	< 24	< 120	200	< 200	–	–	1–6	–	–	B	–	100
Graczyk et al. [114]	A	FINE	S	M, R, U	5 y	S	–	300	< 255	25–299	< 3 min	–	< 6	–	Rect.	B	–	–
Electrical Stimulation for Motor Function, Pain Relief, or other purposes																		
Nashold et al. [39]	CP	Cuff, Button	S	M, R, S, U	< 11 y	O	–	^c^	300	25–100	–	–	–	–	–	B	–	–
Waters et al. [6]	P	Cuff	S	P	< 12 y	M	–	^c^	200	33	–	–	–	–	Rect.	–	–	–
Ben-Menachem et al. [83]	E	Cuff	S	V	14 w	O	–	250–3000	130–500	1–50	30–90	–	–	5–23	–	–	–	–
Elefteriades et al. [5]	Q	Ribbon	S	Ph	< 18 y	M	15–690	100–4600	150	7.2–8.3	0.9–1.3	0.016–0.48	8–12	0.2–0.4	–	–	–	–
Vandoninck et al. [117]	UI	Needle	S	T	< 1 d	O	4000	0–20,000	200	20	–	0.5	0.5	100?	–	–	–	–
Fisher et al. [93]	SCI	Cuff	S	F	3 y	M	160–420	800–2100	200	20	760	–	–	–	–	B	–	–
Abdellaoui et al. [118]	COPD	T	S	–	–	–	6000-18,800	15,000-47,000	400	35	3600	1	1	100	Rect.	B	S	–
Christie et al. [26]	A, SCI	Cuff	S	A, F, Fi, LT, Mu, R, Su, Th, T	< 11 y	M, S	–	100–20,000	1–255	12.5–100	–	–	–	–	–	B	–	–

Subjects: *AB* Able-bodied, *A* Amputee, *COPD* Chronic obstructive pulmonary disease, *CP* Chronic pain due to peripheral nerve injury, *P* Centrally paralyzed ankle dorsiflexor muscle, *Q* Quadriplegia, *SCI* Spinal cord injury, *UI* Urge incontinence

Electrode types: *Cuff*, *LIFE* longitudinal intrafascicular electrode, *Needle*, *Ribbon*, *tf-LIFE4* thin film longitudinal intrafascicular multielectrode, *T* transcutaneous electrode, *TIME* transversal intrafascicular multichannel electrode, *USEA* Utah Slanted Electrode Array

*S* Stimulation, *R* Recording

Electrode Placement: *A* axillary, *F* femoral, *Fi* fibular, *LT* long thoracic, *M* median, *Mu* musculocutaneous, *P* peroneal, *Ph* phrenic, *R* radial, *S* sciatic, *Su* suprascapular, *Th* thoracodorsal, *T* tibial, *U* ulnar

Purpose of Stimulation: *M* motor function, *O* other, *S* sensory feedback

Stimulation Parameters: see Tables 1 and 2

Waveform: Shape: *Rect* Rectangular

Polarity: *M* Monophasic, *B* Bipphasic

Symmetry: *S* Symmetric, *A* Asymmetric

^a^- continuous pattern

^b^ − individually determined, no specification

^c^ − voltage modulation

Earlier publications have suggested the use of stimuli in the range of 50 to 1000 μs [78], or starting as low as 10 μs [38]. The pulse durations mentioned in the modern peripheral nerve stimulation literature are generally below 300 μs (see Table 4). Strength-duration curves show that when considering charge injection, pulses of shorter duration are more effective in eliciting tissue responses. Crago et al. found that narrow, high amplitude pulses are more effective (with less charge) than long, low amplitude pulses for intramuscular and neural stimulation in rats and cats [79]. Butterwick et al. obtained similar results stimulating chick chorioallontoic membranes and retinas, observing a decrease in threshold current density proportional to increasing pulse duration [80]. Similarly, Prado-Guitierrez et al. compared pulse widths of 104 μs and 208 μs in stimulation of the cochlea in guinea pigs [81]. They noted a decrease in current level required for the longer pulse duration, however, this decrease was not proportional to the delivered charge, and therefore longer pulses were deemed less efficient in terms of injected charge. These findings are explained by the non-linearity of the strength-duration relationship as described early on by Lapicque [60].

Modification of the pulse duration can allow for spatial selectivity and steeper recruitment curves [75]. In the case of eliciting somatosensory perception via peripheral nerve stimulation, Tan et al. found that the quality of a sensation can be altered by pulse width modulation [17], and more recently, Graczyk et al. documented the sensory correlates of varying pulse width and frequency [19]. The effect of quality, location, and intensity of elicited percepts when stimulating afferent fibers by varying pulse width in comparison to pulse amplitude have yet to be adequately documented.

### Amplitude

Pulse amplitudes are highly influenced by the electrode placement, as larger target distances require larger stimulation currents [82]. Therefore, extrafascicular electrodes are generally operated at higher amplitudes than their intrafascicular counterparts. Furthermore, a nerve with a larger diameter naturally requires higher amplitudes for the recruitment of all its fibers. Amplitudes of up to 3100 μA have been used safely for stimulation of cat sciatic nerves [45]. In humans, Christie et al. and Vandoninck et al. applied amplitudes of up to 20 mA with cuff and needle electrodes to the fibular and tibial nerves, respectively. Ben-Menachem et al. stimulated the vagus nerve with amplitudes up to 3 mA using cuff electrodes [83]. For intrafascicular electrodes, maximal amplitudes usually lie in the range of 10 to 300 μA for motor and sensory nerves (see Table 4). In regards to safety, Agnew et al. suggested scaling damaging threshold as the ratio to the smallest pulse amplitude required for full recruitment of the alpha component of the compound action potential in the same individual [44].

Overall, one must keep in mind the safety limits of pulse width and amplitude both independently and in conjunction. This is essential during experiment design and clinical applications, to reduce unnecessary risks and optimize efficacy of energy consumption.

### Pulse waveform

Waveform affects both physiological response and safety aspects of nerve stimulation. Pulses can vary in polarity, shape, symmetry, phases, and presence or absence of an interphase delay. In a recent review on restoration of somatosensory feedback via stimulation of peripheral nerves, Pasluosta et al. provided a thorough overview of different waveforms used in the field [84].

A traditional safety restriction placed on a stimulus’ waveform in order to prevent electro-chemical damage is a biphasic, asymmetric, charge-balanced waveform, first introduced by Lilly et al. [85], and commonly referred to as the 'Lilly pulse'. Charge-balanced, or at least biphasic, waveforms are now commonly used (see Table 4) in order to limit any electro-chemical change [77, 86]. In terms of electrode corrosion, however, slightly unbalanced biphasic pulses might be advantageous [2, 77]. In this case, careful consideration must be given to the passive recovery between stimuli (the electrode potential recovery through the inactive stimulator output impedance).

Anodic first pulses have been used in some cases, but cathodic first pulses are the more common practice. The cathodic phase is intended to activate the excitable membrane while the anodic phase is supposed to reverse chemical processes that took place during the initial cathodic phase.

**Fig. 3:**
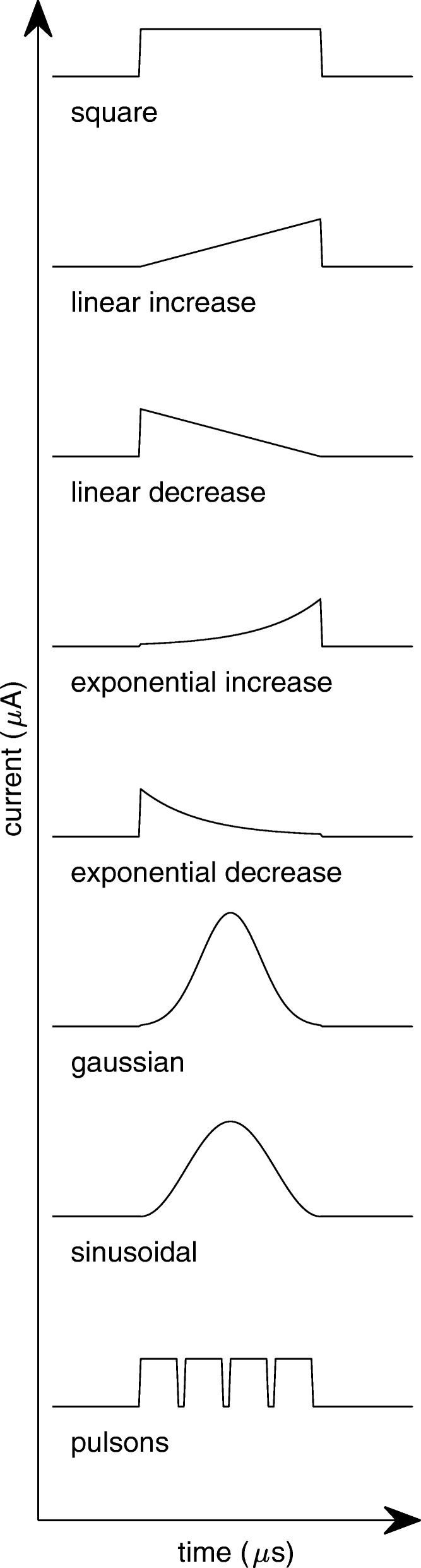
Waveform parameters

Stimulus shape parameters described here correspond to square pulses, because this shape is the most common waveform used to date. Due to the simplicity of generating square pulses, studies were traditionally conducted with this pulse shape. Further work exploring different waveforms is underway (see Fig. 3), however it is mostly limited to computer simulations at present. Non-rectangular waveforms may be less or more effective in eliciting physiological responses. Wessale et al. stimulated human subjects with surface electrodes to examine the effect of rectangular versus exponential pulses [87]. They found that rectangular pulses require slightly lower currents to reach threshold. It was hypothesized that this effect resulted from the phenomenon of accommodation, which entails an elevation of the threshold with slowly rising stimulus slopes. More recently, Sahin and Tie compared linear as well as exponential increase and decrease with rectangular, gaussian and sinusoidal waveforms. Using computational models of nerve membrane potentials, they found the chronaxie to be longer with all non-rectangular pulses. The linear, gaussian and exponential decrease waveforms were most effective in terms of charge injection and lowest threshold charge [88]. Wonsarnpigoon et al. evaluated different waveforms in terms of energy, charge, and power optimization by computational models and in vivo experiments. None of the waveforms—square, rising ramp, rising exponential and decaying exponential—were optimal in terms of all three parameters evaluated. All the reports above suggest that the strength-duration curve can vary depending on the stimulation waveform [89].

Gorman and Mortimer compared square and exponential decay waveforms for the reversal phase and found exponential decay to be more effective in terms of charge injection, as the reversal effect of the exponential decay waveform was lower. A completely novel approach was introduced by Qing et al. suggesting the use of short bursts called pulsons to optimize stimulation in terms of charge required to reach threshold and selectivity [90].

### Inter-phase delay

The inter-phase delay in biphasic pulses is an essential factor, especially when stimulating close to the threshold required for generating action potentials. This delay duration is selected to 1) be long enough to minimize the stimulation threshold, while 2) still being short enough to maintain the anticorrosive effect of the charge recovery phase.

Van den Honert and Mortimer found that introducing an inter-phase delay can lower the stimulation threshold [91]. They observed that up to 100 μs inter-phase duration and amplitude of the recovery phase influenced the degree to which an action potential could be abolished (annihilated after initiation). Gorman and Mortimer confirmed these results, observing steeper recruitment curves (stimulus current vs normalized force) and lower threshold currents with increasing interphase delay [75]. Similarly, Prado-Guitierrez et al. observed that an increase of the inter-phase delay from 8 μs to 58 μs evoked larger compound action potentials, and auditory brain stem responses, when stimulating the auditory nerve in guinea pigs [81]. In early stimulation protocols, inter-phase delays of up to 400 μs were inserted between stimulating and reversal phases [45]. However, little difference between monophasic and biphasic stimuli was noted for delays larger than 80 μs, indicating that the reversing phase has no effect on abolishing action potentials if the inter-phase delay is longer than 80 μs. On the other hand, Merrill et al. recommend the introduction of an inter-phase delay smaller than 100 μs in order to limit electrode corrosion [2]. Therefore, an inter-phase delay of about 80–100 μs can offer an efficient way to reduce threshold currents in biphasic pulses, while still ensuring that the recovery phase starts in time to reverse the electrochemical reactions.

### Frequency

Another point often overlooked is that parameters such as frequency and percentage of effective stimulation time can be damaging by physiologic functional mechanisms. In other words, high frequency pulses can impose an inacceptable level of activity to physiological structures. The resulting damage can be reversible or not and independent of the severity of the overload. Agnew et al. first noted the relevance of pulse frequency when stimulating the peroneal nerve in cats [44]. Their results showed no neural damage after continuous stimulation at 20 Hz for 16 h. However, with otherwise identical parameters, a frequency of 50 Hz resulted in neural damage. Later, McCreery et al. performed more thorough studies on the frequency parameter stimulating the sciatic nerve in cats [92]. For all tested degrees of fiber recruitment, a frequency of 20 Hz proved to be safe, whereas with increasing frequency (50 and 100 Hz) even pulses with smaller currents, and therefore only partial fiber recruitment, resulted in neural damage. McCreery et al. also highlighted the importance of the percentage of ‘effective stimulation time’ in relation to the choice of frequency (further discussed below). In agreement with these findings, Waters et al. safely applied stimuli with a frequency of 33 Hz for a period of up to 12 years to the human peroneal nerve [6], Ben-Menachem et al. used a frequency of typically 30 Hz for 14 weeks in human vagus nerve stimulation [83], Elefteriades et al. applied frequencies of 7.2–8.3 Hz over periods up to 18 years in human phrenic nerves [5], and Fisher et al. stimulated the human femoral nerve with 20 Hz for 3 years [93]. The relatively low frequency tolerance found in the peripheral nerves does not seem to apply as severely to the central nervous system. In stimulation of the cochlear nucleus of guinea pigs, McCreery et al. showed that frequencies up to 100 Hz caused no damage nor stimulation-induced depression of neuronal excitability (SIDNE) after 7 h of continuous stimulation while higher frequencies of 250 and 500 Hz were damaging [94]. Kim et al. stimulated the phrenic nerve of dogs with frequencies of 7 and 35 Hz over periods up to 52 weeks and noted only minimal neural damage, which was presumed to be caused by mechanical restriction rather than the electrical stimulation [43].

Intensity and quality of referred sensations can be encoded by frequency modulation in direct peripheral nerve stimulation [19]. The range of frequencies used in such application has been wide, ranging from up to 300 Hz in initial experiments [95] to 500 Hz in some recent studies [7–9]. However, these experiments were acute and permanent nerve damage was not evaluated although arguably negligible owing to the short duration of the study and experimental sessions. As lower stimulation frequencies are sufficient for other applications (see Table 4), supporting evidence for the safety of long-term electrical stimulation of peripheral nerves only exists for frequencies below 30 Hz at present.

### Percentage of effective stimulation time

The term ‘duty cycle’ has been used in the past to describe the percentage of 'ON' versus 'OFF' stimulation time [44, 94, 96]. For instance, stimulation of 5 s (‘ON’), follow by a pause of 5 s (‘OFF’) before engaging in stimulation again was described as a 50% duty cycle by dividing the ‘ON’ time (5 s) by the overall time (10 s). However, in engineering, the ‘duty cycle’ is usually defined over a single pulse duration (D + D’), rather than to the total period of stimulation as aforementioned. For instance, a duty cycle of 50% would normally imply that a pulse with a period of 1.0 s is active for 0.5 s, regardless of how long a stimulation is being delivered. In other words, a 100% duty cycle would be equivalent to DC stimulation (or to a pulse duration equal to the pulse period), and 0% duty cycle would be equivalent to no or 'OFF' stimulation. In order to avoid ambiguity between these two definitions, in this article we use the term ‘percentage of effective stimulation time’ to refer to the former (O=S/P). Train rate (R), is a equivalent to stimulation pulse frequency (F) when the total application time is equivalent to the effective stimulation time (S=P), see Tables 2 and 3.

It has been suggested that by decreasing the percentage of effective stimulation time one can safely increase other stimulation parameters. Agnew et al. showed that with 50% effective stimulation time, it is possible to stimulate a cat peroneal nerve at higher frequencies than 50 Hz, and for a period of 16 h, while causing considerably less damage to the nerve than at 100% effective stimulation time [44]. Tykocinski et al. observed a smaller decrease in excitability and faster recovery after stimulation of the auditory nerve in guinea pigs when using 50% effective stimulation time compared to continuous stimulation [96].

The impact of the percentage of effective stimulation time in human peripheral nerve stimulation is rarely documented in the literature for both acute and chronic applications. In an early acute study, Anani et al. stimulated median and radial nerves of able-bodied subjects with an effective stimulation time of 17%. Although threshold changes made an up-regulation of parameters necessary in some cases, no indication of permanent damage was reported [97]. Elefteriades et al. stimulated the phrenic nerve for 8 to 12 h per day with 17 to 26% of effective stimulation time and found constant threshold currents over time periods of up to 18 years [5]. Similarly, vagus nerve stimulation is commonly applied with low percentages of effective stimulation time of 9–27% [98].

McCreery et al. suggested the use of a parameter they called ‘average frequency’, equal to stimulus frequency times the percentage of effective stimulation time, as a means of predicting SIDNE, which is considered an early stage of neural damage [94]. When stimulating the cochlear nucleus in cats for 7 h at higher overall average frequencies above 100 Hz, they noted an increase in the threshold of the evoked response proportional to the average frequency, indicating this parameter is more meaningful than either frequency or percentage of effective stimulation time alone. In their study, only one frequency at a time was employed, followed by a period of no stimulation. Therefore, speaking of an ‘average’ could be misleading since an average normally corresponds to variable values over the number of values. Consequently, the term ‘weighted frequency of effective stimulation time’ is proposed as an alternative to ‘average frequency’ (see Tables 2 and 3). The pioneering work of McCreery et al. has not been continued nor expanded upon to other frequencies or neural tissues.

### Stimulation application time and treatment duration

In this article, the stimulation application time is considered as the time per day during which stimulation is active, whereas treatment duration is the overall duration an electrode was implanted and active. As discussed previously, the length of stimulation application has been shown to influence the degree of induced neural damage. Specifically, Agnew et al. noted that it is possible to stimulate safely with identical parameters if the duration of stimulation was decreased from 16 h to 4 h [44]. The duration of continued stimulation rarely exceeded 8 h in animal studies, making it difficult to draw conclusions on the length of time one can safely stimulate peripheral nerves based on these experiments. In human studies, stimulation application length in acute experiments is commonly below 6 h, and normally intermittent (see Table 4). Chronic but intermittent stimulation had been reported successful for therapy periods up to 18 years [5]. However, information on the safety of chronic and uninterrupted peripheral nerve stimulation is absent, and yet needed for applications such as restoring proprioception and sensory feedback capabilities of limb prostheses.

## Transferability

Due to the lack of experimental data on safety restrictions specific to human peripheral nerve stimulation, researchers often extrapolate from findings about other nerves and/or animal studies. A brief review of the appropriateness of such a practice is given in this section.

### Transferability between species

Ethical considerations often prevent testing safety limitations in humans. Consequently, a large part of the literature and of our knowledge about neural stimulation is based on animal studies mostly conducted in the 1980s and 1990s [43–45, 62–64, 75, 92, 94, 96, 99, 100]. Biostability of electrodes as well as passive harmlessness are similar in animals and humans. In an early review, Agnew and McCreery argued that when extrapolating stimulation parameters from animal studies to humans, physiological parameters such as diameter of the stimulated nerve should be considered above other factors [1].

Transferability between species is generally admitted for passive and active aspects of biostability and passive aspects of harmlessness. However, there are sometimes diverging reactions to electrical stimulation due to functional physiological differences as well as easily identifiable parameters such as conduction velocity, fiber diameter, axon count, or myelination. Transferability should be considered with caution when these parameters diverge too significantly.

### Transferability between tissue types

Studies on stimulation of the peripheral nervous system are not always a suitable reference for stimulation of the central nervous system or muscle tissue, and vice versa. However, passive and active biostability are expected to show some similarity between various tissue types, and are highly comparable if the temperature and composition of the surroundings are similar.

Previous reviews have highlighted that physiological differences between the brain and peripheral nerves explain the differences in the range of electrical stimulation parameters used in both situations [25, 101]. In transcutaneous peripheral stimulation, pulse durations observed in the literature are longer (up to 400 μs) than those given for implanted electrodes (see Table 4). Pulse frequencies used in skin surface stimulation are generally in the range of 8 to 100 Hz [102], and therefore below frequency ranges commonly observed in neural stimulation (see Table 4). Use of higher frequencies of 100 Hz and 200 Hz has shown to be viable for stimulation of the auditory nerve [96].

In summary, physiological characteristic differences must be considered when comparing active harmlessness, whereas passive harmlessness and biostability characteristics in general are expected to be relatively similar.

## Diversity in the peripheral nervous system

Stimulation safety limits cannot be applied as a global concept to neural tissue due to the diversity of cells forming it [103]. Peripheral nerves have a variety of fibers with different physiology and function, as well as Schwann cells, blood vessels, and various supporting cells [42, 104–106]. All these cells are potentially sensitive to electrostimulation in different ways leading to diverse consequences.

Neural stimulation has more effects than the mere initiation of an ‘action potential’ transmitted to higher physiological structures. Some of these effects could be referred to as trophic effects of neural stimulation. Collateral sprouting in motor nerves and regeneration in sensory nerves are modulated by the neural activity [107–110]. Similar mechanisms probably exist throughout the nervous system and some forms of cerebral plasticity can be interpreted in this context [111]. However, the underlying mechanisms remain poorly known. The SIDNE phenomenon and the demonstration that ‘overstimulation’ can kill a nerve [44] can also be considered as a trophic mechanism [112]. Although this has never been demonstrated, it would seem logical that safe stimulation should not impose much more activation to the nerve than its functional physiological level. This limit can vary between different nerves and should thus be investigated for each neural structure and nerve type separately.

Maximal tolerated stimulation pulse frequencies vary for different neural structures as demonstrated in a number of publications including the relatively low stimulus frequency tolerance of motor nerves when compared to examples in the central nervous system [44, 65, 92]. Not only the frequency but the effect of each stimulus parameters must be considered. There is thus a need for specific chronic safety studies in various neural structures considering all stimulus parameters. These studies still leave open the question of validity for humans. Numerous studies have already been published including several reviews [1, 4]. Unfortunately, the available data still fall short of covering the topic in any systematic way, and it must be pointed out that at best, the limits proposed today are mere extrapolations.

## Classification of neural damage

Evidently, knowledge about the safety limits of stimulation parameters is rather limited, especially for peripheral nerves in humans. Although electrode design has significantly improved and conditions for passive biostability and harmlessness are generally agreed upon, a high degree of uncertainty about the effects of electrical stimulation remains.

Inflammation is always present after chronic implantation. This can be inactive, leaving fibrous tissue which can for example shield the nerve fibers from the stimulation current. Resulting variations in impedances and stimulation thresholds seem to stabilize 8 weeks post implantation [51]. If the inflammatory reaction remains active, various molecular factors that can directly affect the functionality of neural tissue are being released.

A major issue in comparing different studies is the lack of a common classification of reversible and irreversible neural damage. Until now, neural damage has been evaluated in terms of evoked action potential and functional performance in living subjects, as well as by post-mortem histology. Local damage in peripheral nerves can occur through several mechanisms, resulting mainly in axonal degeneration and demyelination. These damages could be reversible if no further stimulation is applied [54, 113].

Several techniques have been used to evaluate the degree of damage. Agnew et al. recorded the amplitude of the alpha component of the compound action potential at different current intensities before and after stimulation of the peroneal nerve in cats [44]. However, no direct correlation was found between amplitude changes and the percentage of degenerated axons. McCreery et al. used a similar amplitude-based principle to evaluate SIDNE in stimulation of the cat’s posteroventral cochlear nucleus. They showed a correlation between the level of SIDNE and the pulse frequency and stimulation duration, respectively, but no injury could be detected on histological evaluation [94]. Adaptation mechanisms taking place during electrical stimulation of peripheral nerves as described by Graczyk et al. [114] may also have an effect on what McCreery et al. described as SIDNE. Potential connections must be explored in the future. Demyelination should also result in slowing of the nerve conduction velocity if the affected nerve section is long enough for the measurement to be performed.

Peripheral nerves, like brain tissue, contain not only various types of axons but also other important cells such as the Schwann cells, immune cells, and vascular structures with blood-nerve barrier, all in an organized anatomical arrangement. Considering this complexity, it is perhaps not surprising that different evaluation parameters yield different results. A study on the effect of the individual stimulation parameters on specific physiological characteristics is necessary to yield a transferable understanding of damaging limits. In this context, the functional significance and reversibility of any observed change must also be considered, because tissue physiology participates actively in the implantation process. There is therefore still a need for better discrimination and classification of the various actors and targets of peripheral nerve stimulation safety limits.

## Conclusion

In this work, we summarized current knowledge on safe stimulation protocols in peripheral nerves and discussed relevant concerns. Currently, extraneural electrodes have proven safe for chronic applications while invasiveness and long-term stability of intraneural electrode remain challenging for permanent implantation. Safety limits of stimulation parameters are still predominantly described in terms of electric charge. The popular Shannon equation may provide some guidance toward elucidating safety limits, however the same equation points to the fact that acceptable limits cannot be normalized to the electrode contact size. Consequently, normative data cannot be applied to electrodes of different sizes. Regarding pulse width and amplitude, short pulses require lower electric charges to elicit action potentials, and therefore short pulses should be preferred whenever the necessary higher current amplitudes are technically feasible. The only available study about the allowable stimulation frequency in chronic peripheral nerve stimulation suggests that 50 Hz is a maximal limit. However, with a reduced percentage of effective stimulation time, higher frequencies might still be safe as well. Long-term studies should further investigate this issue. Furthermore, the use of different pulse shapes could improve efficiency as suggested by computational models and in-vitro experiments, but in-vivo comparative studies are still lacking. Direct individual functional monitoring, as suggested by Agnew et al. in 1989, could offer a useful alternative to published safety limit values for the clinical determination of electric stimulation parameters [44], and future safety studies should consider the diversity of cells in peripheral nerves and how these are affected by electrical stimulation. The reactions of the different neural structures to an implant and to electrical stimulation can cause structural and functional changes over time. In addition, the therapeutic use of neuromodulation can induce phenomena such as central plasticity and peripheral axonal growth or collateral compensation. Time is thus an essential parameter and there is a need for chronic follow up studies.

A major limitation when performing this review was the lack of systematic documentation of stimulation parameters in the literature. We hope this paper will help drawing more attention to these aspects and help to standardize the reporting of stimulation protocols (Tables 2 and 3). The aim of such a parameter list is to allow for effective comparison and meta-analysis that draw more meaningful and broader conclusions about the safety aspects of neural stimulation.
