# Correction to: Safety of long-term electrical peripheral nerve stimulation: review of the state of the art

**DOI:** 10.1186/s12984-020-00695-1

**Published:** 2020-06-15

**Authors:** Clara Günter, Jean Delbeke, Max Ortiz-Catalan

**Affiliations:** 1 Biomechatronics and Neurorehabilitation Laboratory, Department of Electrical Engineering, Chalmers University of Technology, 41296 Gothenburg, Sweden; 2 LCEN3, Department of Neurology, Institute of Neuroscience, Ghent University, C. Heymanslaan, 10, 9000 Ghent, Belgium; 3 Integrum AB, Krokslätts Fabriker 50, 43137 Mölndal, Sweden

**Correction to: J NeuroEngineering Rehabil 16, 13 (2019)**

**https://doi.org/10.1186/s12984-018-0474-8**

The original article [1] contains an error in Table 3 whereby derivation for ‘T’ should be ‘N/F’ instead of ‘S/P’.
